# External Deformation Monitoring and Improved Partial Least Squares Data Analysis Methods of High Core Rock-Fill Dam (HCRFD)

**DOI:** 10.3390/s20020444

**Published:** 2020-01-13

**Authors:** Xiang Cheng, Qingquan Li, Wei Zhou, Zhiwei Zhou

**Affiliations:** 1 State Key Laboratory of Surveying, Mapping and Remote Sensing Information Engineering, Wuhan University, Wuhan 430072, China; xiang.cheng@whu.edu.cn (X.C.); liqq@szu.edu.cn (Q.L.); 2 Key Laboratory of Geo-Environmental Surveillance in the Maritime and Marine Zones, National Mapping and Geographic Information Bureau, Shenzhen University, Shenzhen 518061, China; 3 State Key Laboratory of Water Resources and Hydropower Engineering Science, Wuhan University, Wuhan 430072, China; 4 State Key Laboratory of Geodesy and Earth’s Dynamics, Institute of Geodesy and Geophysics, Chinese Academy of Sciences, Wuhan 430077, China; Zhiwei.Zhou@whigg.ac.cn

**Keywords:** angle forward intersection method, high core rock-fill dams (HCRFD), improved partial least squares method, machine learning, geodetic control network, total station

## Abstract

External deformation monitoring of high core rock-fill dams (HCRFDs) is an important and difficult part of safety monitoring. The traditional method of external deformation monitoring and data analysis for HCRFDs is to use a total station for small angle observations and establish a regression model to analyze the results. However, the small angle method has low accuracy and a low automation degree, and there is multicollinearity between the independent variables, which affects the parameter estimation and leads to the failure of model establishment. The angle forward intersection method is adopted in this paper for observation, and an improved partial least squares method (IPLS) is proposed to eliminate the multicollinearity of the independent variables. Compared to the traditional method, the improved observation method exhibits high accuracy and a high automation degree. The new data analysis method can not only eliminate multicollinearity but also improve the interpretation ability of the model. The data from the initial stage of water storage shows that the displacement increases with the increase in the upstream water level and time, and the speed of water storage is proportional to the displacement. The water level and time are the main influencing factors. This conclusion provides a theoretical basis for reservoir management departments to control water levels and gate opening and closing. The method in this paper can be applied to arch dams, gravity dams, and other types of waterpower engineering systems.

## 1. Introduction

High core rock-fill dams (HCRFDs) are inexpensive and simple to construct and can be constructed from locally sourced materials. They possess good adaptabilities to dam foundation conditions, make full use of construction excavation materials, and exhibit good seismic performance. They play an essential role in the development of water resources worldwide. HCRFDs are earth and rock dams that are widely used in countries all over the world at present [1]. Once the high dam is damaged, it can cause severe safety accidents that threaten human lives, property, and the environment. Therefore, the safety monitoring of HCRFDs is quite necessary [2]. The safety monitoring of HCRFDs include internal and external methods. Some studies have shown the use of a horizontal displacement meter to measure internal horizontal displacement and a water-pipe sedimentation meter to measure internal vertical displacement [3]; however, when a dam height is over 300 m, these internal sensors are useless due to large deformation over time [4]. The pendulum is an effective method to measure the internal displacement and is usually used in gravity dams and arch dams. Regarding HCRFDs, this sensor is not used [5]. All internal deformation monitoring methods are relative measurement methods.

External deformation monitoring of HCRFDs is a priority for safety monitoring, and it also is the issue about which managers are most concerned. There are two types of methods for external deformation monitoring: contact and non-contact methods. The non-contact methods mainly include interferometry synthetic aperture radar (INSAR) [6] and laser light detection and ranging (LIDAR) [7]. Non-contact monitoring is not considered in this paper. The traditional contact methods include using a total station and global navigation satellite system (GNSS) [8,9]. GNSS antenna should be installed at every measurement point except the choke ring GNSS antenna at the control point. The cost of GNSS is high and this article is focused on total station. The total station small angle method is one of the traditional methods used for deformation monitoring [10]. Using this simple method, a total station is set up on datum points on the end of a collimation line, and the points on the collimation line are observed manually by the small angle method. However, measurements are obtained one-by-one along the collimation line, and unified automatic measurements cannot be achieved. Furthermore, long measurement times and low automation levels are shortcomings of this method [11]. Additionally, the small angle method is limited by the measuring distance and angle, and when the measured distance is 100 m, the angle measurement accuracy is 2″, and the mean square error is about 2 mm, the accuracy is low. Some studies adopt the method of a geodetic control network for deformation monitoring [12,13]. During these studies, one total station is used to observe the control network manually, adjust the control network and correct the displacement value of the working basis. However, the measurement time is too long, and the measurement period is half a year, so it is impossible to observe the dam collimation line automatically in real time and dam deformation cannot be obtained in time.

The external deformation displacement of an HCRFD is affected by the reservoir water level, rainfall capacity, time, temperature, atmospheric pressure and downstream water level during the storage period [14]. After the measurements are completed, the correlation between the displacement and the influencing factors must be analyzed. The traditional data analysis method is to establish a regression equation between the displacement and the relevant variables [15]. However, there may be multicollinearity between the independent variables, which affects the parameter estimation and can result in failure of the regression model establishment [16]. Seen in the field of machine learning, the methods for eliminating multicollinearities include the principal component analysis (PCA) and partial least squares (PLS) methods [17]. Both the PCA and PLS methods extract the principal components from the set of independent variables, and the extracted principal components are linearly independent, which resolves the issue of the multicollinearity of the independent variables [18]. However, when the extracted components contain significant amounts of information that is unrelated to the dependent variables, the results of the PCA and PLS methods are not satisfactory [19]. Some studies use the PCA and the back propagation (BP) fusion method to analyze dam monitoring data. They use PCA to solve the multicollinearity and then put data into a BP neural network as the input layer [20]. Although this method can eliminate multicollinearity, it only depends on independent variables, and the explanation of dependent variables will diminish just as PCA. Meanwhile, the mode used in this paper is only used for arch dams not for the core rock-fill dams. Some studies use a partial least squares regression (PLSR) method for the dam displacement monitoring and they use the PLS to establish the model directly [21], while not considering the situation of the extracted components containing significant amounts of information that is unrelated to the dependent variables.

Aiming at the issues of the external deformation monitoring methods and data analysis of traditional HCRFDs, an angle forward intersection method using a total station and an improved partial least squares (IPLS) data analysis method is presented in this paper. The angle forward intersection method sets two control points on the left and right banks of the dam. The control points and the measurement points of HCRFD form a local network. The coordinate values of the control points are obtained by adjustment of the geodetic control network [22]. Through the comparison of the methods, the accuracy of the small angle and angle forward intersection methods are analyzed in detail, revealing that the new observation method is effective and advanced. By comparing and analyzing the advantages and disadvantages of the multiple linear regression (MLR), PCA, and PLS methods for handling multicollinearities, a new data analysis method—the IPLS method—is proposed. Taking the measured initial storage data of the Nuozhadu HCRFD as an example, the practicability of the new observation and data analysis methods are studied to explore the relationship between the HCRFD downstream rock-fill displacement and the independent variable factors during initial storage. This conclusion provides a theoretical basis for reservoir management departments to control water levels and gate opening and closing. This method will provide technical assistance for reservoir operation administrative departments and establish a research basis and guidance for arch dams, gravity dams, and other types of water-powered engineering structures.

The main content of the article is as follows. The main research area and the traditional methods of observation and data analysis are analyzed in Section 2. A new observation method is proposed in Section 3 and compared with the traditional method. A new data analysis method is proposed in Section 4 and compared with the traditional method. The results, field measurement data, and displacement of the initial storage change are analyzed in Section 5. Finally, a discussion and conclusions are presented in Section 6.

## 2. Research Area and Traditional Method Exposition

### 2.1. Research Area

**Figure 1:**
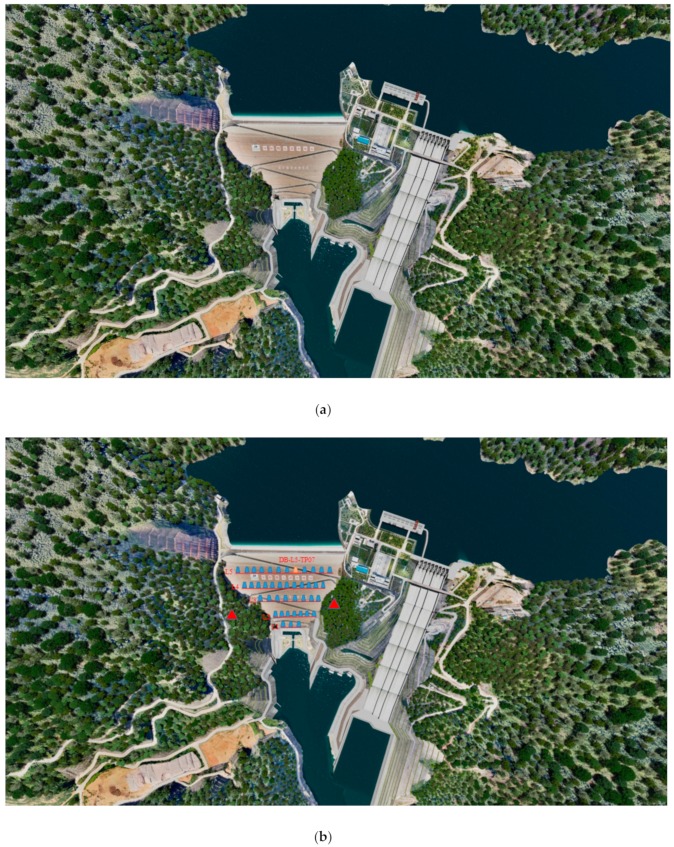
(**a**) Schematic diagram of Nuozhadu dam (Top view). (**b**) Collimation lines and typical measuring points. Five collimation lines (L1–L5) were established on the surface of the downstream dam slope to monitor the surface deformation of the rock-fill body, and the typical measurement point DB-L5-TP-07 was selected as an example.

The Nuozhadu high core rock-fill dam (HCRFD) was the research object (As is shown in Figure 1). The Nuozhadu hydropower station is located on the main stream of the Lancang River at the junction of the Cuiyun district of Simao city and Lancang County, Yunnan province (the dam site is between the Kanjie River and Huoshao village ditch). This dam is the fifth step of eight planning steps for the middle and lower reaches of the Lancang River. The project is a large (1) type first class project, and the permanent main hydraulic structures are grade 1 buildings. The core wall rock-fill dam crest is 627.87 m long, 18 m wide, and 821.5 m high at the crest (the sea level as the elevation datum), and the maximum dam height is 261.5 m. This dam is the third largest core wall rock-fill dam in the world and the first in Asia [23]. We focused on the surface deformation of the downstream rock-fill body and observed the collimation lines L1–L5 of the downstream rock-fill body using a total station. Data collected from all the measuring points along L1–L5 were adopted for the modeling, and the displacements of the measuring points of L5 were larger than those of the measurement points of the other collimation lines. The typical measurement point DB-L5-TP-07 of L5 is used as an example for the data analysis, and the initial storage period from 2013-7-1 to 2013-10-21 was studied.

### 2.2. Traditional Observation Methods

**Figure 2:**
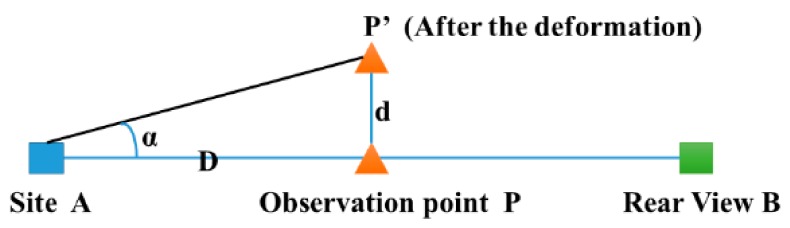
Schematic diagram of the measurement setup.

The traditional observation method is to accurately measure the small angle and the distance between the set station and the observation point using the total station. The principle of observation is shown in Figure 2 where A and B are reference points. The total station is located at point A, and the prism is located at point B. P is the monitoring point, and P’ is the monitoring point after deformation. The angle from total station to the monitoring point is α.

The displacement of point P is denoted as *d* and expressed as follows:

(1) d=D⋅tanα≈D⋅α/ρ

where tanα≈α, because α is small, D is the distance between the total station and point P, and ρ is the conversion parameter with the value is 206,265 (1 radian is about 206,265″, ρ = 60 * 60 * 180/π ≈ 206265).

This is a simple and commonly used method for monitoring the external deformation of a high core rock-fill dam (HCRFD). However, this method requires stations and rear viewpoints to be established one-by-one along each collimation line from L1 to L5. Furthermore, the observations can only be made manually and, thus, the observation efficiency is low. We can see from Equation (1) that the accuracy of the horizontal displacement is affected by the observation error of the observation distance D and horizontal angle α. Because there are no redundant observations, the accuracy is low.

## 3. Improvement of the Observation Method

### 3.1. Angle Forward Intersection Method

**Figure 3:**
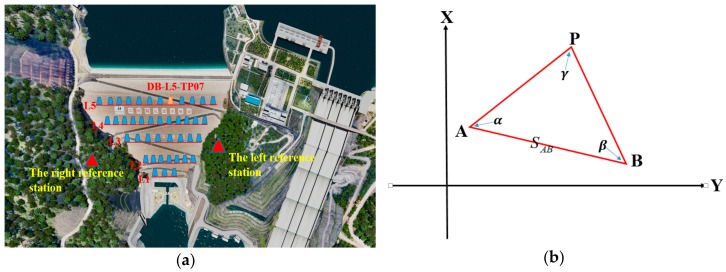
(**a**) Forward intersection method schematic diagram. (**b**) The known conditions and unknown points of the forward intersection method.

During this study, an improved method based on total station was adopted—the angle forward intersection method. The schematic diagram for this method is shown in Figure 3a,b.

Unlike the small-angle method, which requires setting working basis points on each sight line, the forward intersection method sets working basis points A and B at fixed positions on the left and right banks of the dam. Two total stations were set up at points A and B, with point P as the monitoring point and location of the prism. The coordinates of the working basis points A and B are (*X_A_*, *Y_A_*) and (*X_B_*, *Y_B_*), respectively, and the distance between them is $S_{AB}$. The observation angles at points A and B are α and β, respectively. Point P is the monitoring point with coordinates $\left(X_{p},Y_{p}\right)$.

The distance between points A and P is denoted as $S_{AP}$. $\alpha_{AP}$ and $\alpha_{AB}$ are the coordinate azimuths of AP and AB, respectively.

The expressions for the coordinates of point P [24]:

(2) $$\left\{\begin{array}{c} X_{P}=\frac{X_{A}\cdot \cot\beta +X_{B}\cdot \cot\alpha +(Y_{B}-Y_{A})}{\cot\alpha +\cot\beta }\\ Y_{P}=\frac{Y_{A}\cdot \cot\beta +Y_{B}\cdot \cot\alpha -(X_{B}-X_{A})}{\cot\alpha +\cot\beta } \end{array}\right.$$

Equation (2) is used to calculate the coordinates of point *P* in the angle forward intersection method. Added to the two working basis points *A* and *B*, three working basis points can also be set. The redundancy of the observations can be improved by increasing the number of working basis points. Added to the high accuracy and redundant observations, this method can be used to obtain measurements automatically, which improves not only the accuracy of the observations but also the efficiency.

### 3.2. Accuracy Comparison of Observation Methods

Equation (1) shows that the displacement accuracy of the small-angle observation method is affected by observation errors of the observation distance D and horizontal angle α. The horizontal distance D can be used as a fixed value after the observation, and the displacement accuracy is only affected by the horizontal angle α. So, the error in the observation can be calculated using the following equation [25]:

(3) $$m_{d}=m_{\alpha }\cdot D/\rho$$

where $m_{d}$ is the mean square error, $m_{\alpha }$ is the angular accuracy, D is the horizontal distance, and is a constant.

**Figure 4:**
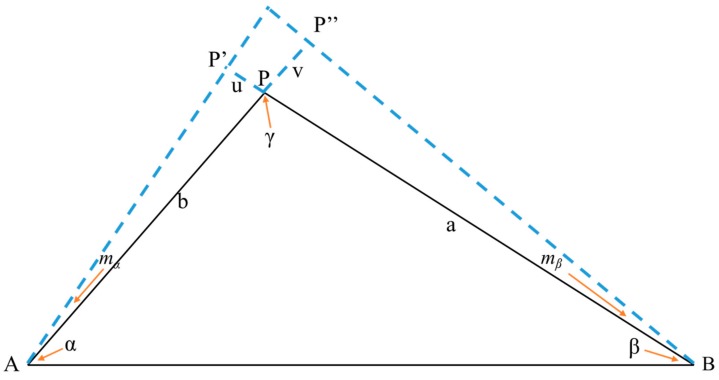
Measurement accuracy derivation schematic diagram. The control points are A and B respectively.

The accuracy of the angle forward intersection method is derived in detail below based on Figure 4. A and B are selected points, and the distance between them is $S_{AB}$_._ α and β are observation angles, γ is the intersection angle, and P is the observation point. $m_{\alpha }$ and $m_{\beta }$ are the observation errors of α and β, respectively. When there is no error in angle β, the error of angle α will cause the displacement of point P in the direction of BP to be u (PP′). When there is no error in angle α, the error of angle β will cause the displacement of point P in the direction of AP to be v (PP″).

**Figure 5:**
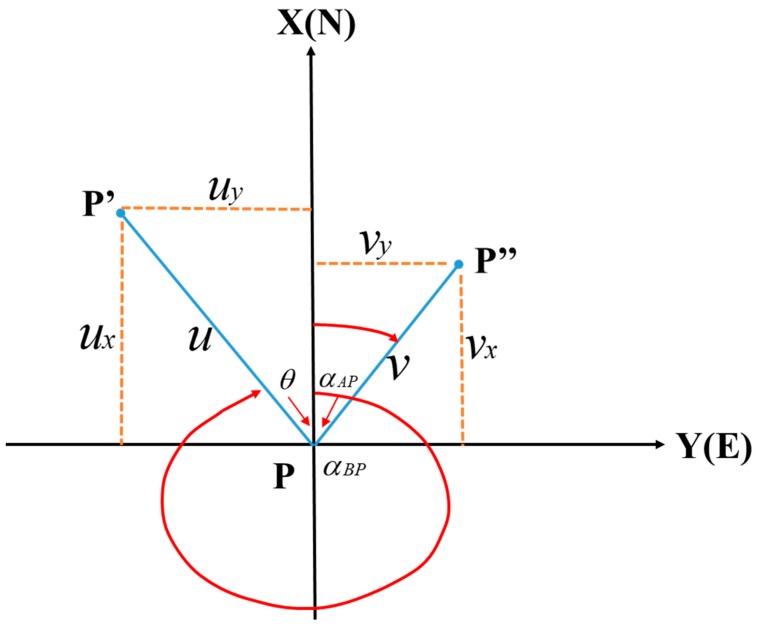
Schematic diagram of axis decomposition.

Generally, α and β are observed with equal precision and, thus, we assume that $m_{\alpha }=m_{\beta }=m$. The x- and y-components of u and v are shown in Figure 5. A rectangular coordinate system is established with P as the origin. The components of u and v on the X- and Y-axes of the coordinate system are $u_{x}$, $u_{y}$, $v_{x}$ and $v_{y}$, and the coordinate azimuths are *α_BP_* and *α_AP_*.

The following relations can be obtained based on Figure 5:

(4) $$\left.\begin{array}{l} u_{x}=u*\cos(2\pi -\alpha_{BP})=u*\sin\alpha_{BP}\\ u_{y}=u*\sin(2\pi -\alpha_{BP})=u*\cos\alpha_{BP}\\ v_{x}=v*\sin\alpha_{AP}\\ v_{y}=v*\sin\alpha_{AP} \end{array}\right\}$$

According to Equation (4), the mean squared errors in the horizontal and vertical coordinates of point P are as follows:

(5) $$m_{x}=\sqrt{u_{x}{}^{2}+v_{x}{}^{2}}=\frac{m}{\rho }\cdot \frac{\sqrt{a^{2}\cos^{2}\alpha_{AP}+b^{2}\cos^{2}\alpha_{BP}}}{\sin\gamma }$$

(6) $$m_{y}=\sqrt{u_{y}{}^{2}+v_{y}{}^{2}}=\frac{m}{\rho }\cdot \frac{\sqrt{a^{2}\sin^{2}\alpha_{AP}+b^{2}\sin^{2}\alpha_{BP}}}{\sin\gamma }$$

Therefore, the mean squared error of point P is as follows:

(7) $$m_{P}=\sqrt{m_{x}{}^{2}+m_{y}{}^{2}}=\frac{m}{\rho }\cdot \frac{\sqrt{a^{2}+b^{2}}}{\sin\gamma }$$

We obtain the following:

(8) $$m_{P}=\frac{m}{\rho }\cdot \frac{S_{AB}}{\sin\gamma }\cdot \sqrt{\sin^{2}\alpha +\sin^{2}\beta }$$

Equation (8) is the accuracy estimation equation for the angle forward intersection method. Based on the comparison of Equations (8) and (3), we assume that distances D and *S_AB_* are equal. When the intersection angle γ≥90°, $M_{p}<m_{d}$ and the accuracy of the angle forward intersection method is higher than that of the small-angle method. When the intersection angle γ > 120°, however, the error will increase and Mp is maybe greater than $m_{d}$. Therefore, when we establish reference stations A and B, γ should be the value between 90° and 120°.

## 4. Improvement of the Data Analysis Method

### 4.1. Review of Existing Methods

Due to the multicollinearity between the independent variables in multiple linear regressions, PCA and PLS regressions have been proposed previously. The main idea of PCA is to calculate the eigenvalues and eigenvectors from the matrix composed of the correlation coefficients of the independent variables and extract the principal components based on the cumulative contribution rate. The extracted principal component is linearly independent and solves the issues that arise from the multicollinearity of the independent variables [26]. The idea of partial least squares regression is to extract the first principal component from the independent and dependent variables, calculate the model accuracy, and repeat the extraction until the accuracy meets the requirements, and the extracted principal component is linearly independent, which also solves the issues arising from the multicollinearity of the independent variables [27]. PCA focuses on the independent variables, and consequently, the interpretation of the dependent variables diminishes. The PLS method not only depends on the independent variables but also the dependent variables [28]. Thus, the interpretation of the dependent variables is improved [29]. However, PLS regression fails when the explanatory matrix formed by independent variables contains a large amount of information unrelated to the dependent variables [30].

Some studies use a PCA and BP fusion method to analyze dam monitoring data. They use PCA to solve the multicollinearity and then put data into the BP neural network as the input layer. Although this method can eliminate multicollinearity, it only depends on independent variables, and the explanation of dependent variables will diminish just as PCA. Meanwhile, the mode used in this paper is only used for arch dams. Some studies use a PLSR method for the dam displacement monitoring, they use the PLS to establish the model directly, not considering the situation of the extracted components containing significant amounts of information that is unrelated to the dependent variables.

To address the failure of the PCA and PLS regression, we propose an improved partial least squares (IPLS) method, based on an orthogonal projection, which eliminates the information that is irrelevant to the dependent variables.

### 4.2. Improved Partial Least Squares Algorithm (IPLS)

The core of improved partial least squares (IPLS) is to eliminate the components that are irrelevant to the dependent variables from the interpretation matrix composed of independent variables using an orthogonal projection. After processing, the interpretation matrix does not contain information that is irrelevant to dependent variables, and partial least squares regression modeling can be used. More specifically, we remove the vectors that are orthogonal to the dependent variable Y and are composed of the interpretation matrix X. The detailed derivation of the algorithm is as follows.

**Figure 6:**
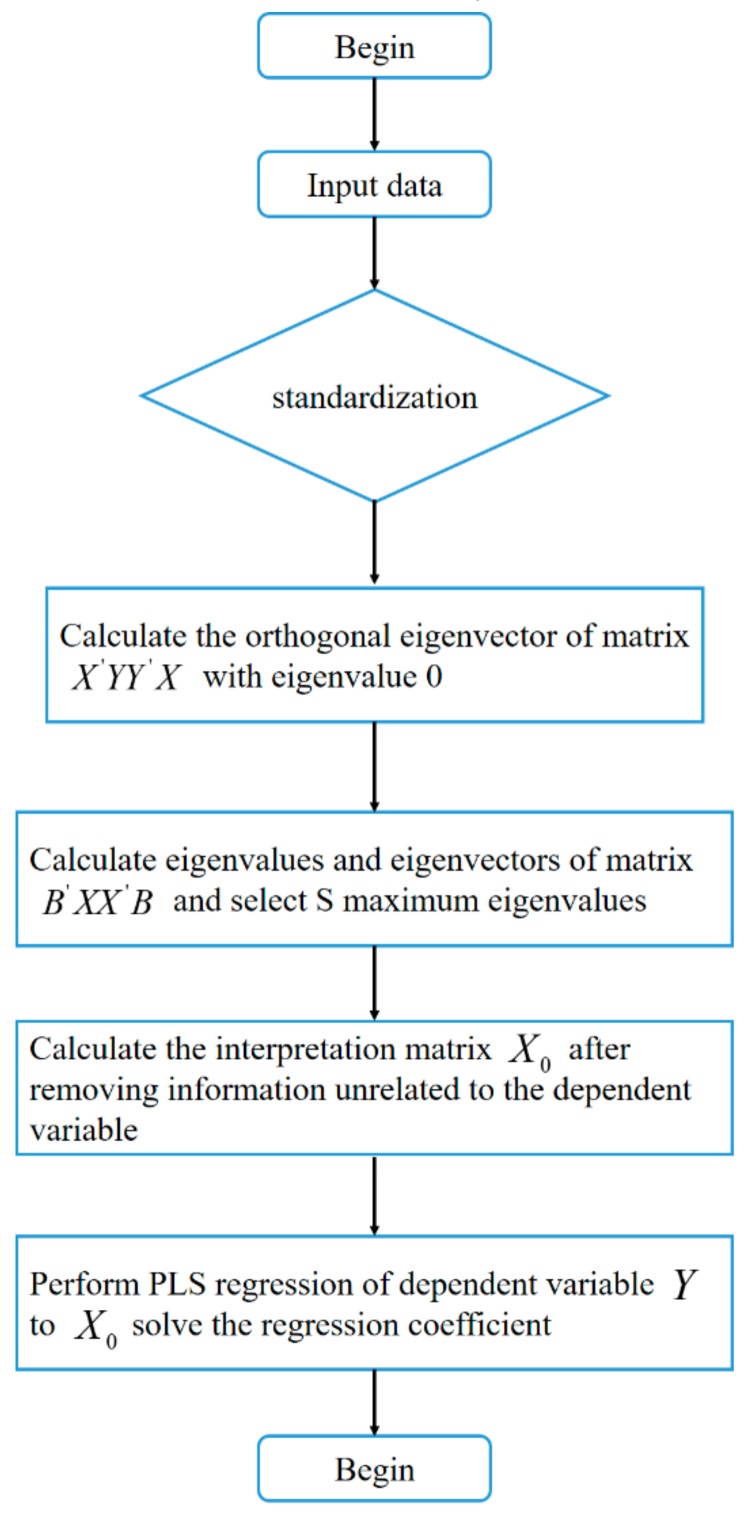
Schematic diagram of axis decomposition.

1. Y is set as an n×1 single dependent variable, X is set as an n×p independent variable interpretation matrix, and both have been standardized. The matrix X′YY′X is a square matrix of order P, which has p−1 orthogonal eigenvectors with eigenvalues of 0. Furthermore, B = $\left(b_{1},b_{2},\cdots ,b_{p-1}\right)$.
2. According to Step 1, $b_{j}(j=1,2,\cdots p-1)$ is an eigenvector with an eigenvalue of 0. Therefore,
  (9) $$X\prime YY\prime Xb_{j}=0,Y\prime Xb_{j}=0$$
  We want to remove the vector that is orthogonal to the dependent variable Y and consists of the interpretation matrix X. Conveniently, this vector is denoted as Xr, and the following relation is satisfied:
  (10) Y′Xr=0
  We obtain the following equation from Equations (9) and (10):
  (11) $$Y\prime Xb_{j}=Y\prime Xr=0$$
  Equation (11) shows that r can be represented by a linear combination of B, which is expressed as follows:
  (12) r=Ba
  Substituting Equation (12) into (11) yields the following relation:
  (13) Y′XBa=0
3. The irrelevant information of the dependent variable Y is eliminated by determining the vector with the maximum variance of *XBa* in Equation (13), i.e., by finding the eigenvector corresponding to the largest eigenvalue in *B′X′XB*. We set $A=\left(a_{1},a_{2},\cdots a_{s}\right)$, (s<p−1) and H=XBA. Thus, H is the information that is independent of Y in the matrix X.
4. The orthogonal projection operator of the interpretation matrix X on H is $P=H{\left(H^{\prime }H\right)}^{-1}H^{\prime }$. X is projected onto the orthogonal complement of H, that is, the interpretation matrix that is irrelevant to the dependent variable is removed:
  (14) $$X_{0}=X-PX$$
5. $X_{0}$ replaces the original interpretation matrix X, and a partial least squares regression is used to determine the relationship between Y and $X_{0}$. The algorithm flow is shown in Figure 6.

## 5. Results

When the initial storage of the high rock-fill dam is too fast or slow, there will be some special conditions such as large leakage, dam top cracking and so on [31]. This phenomenon is closely related to the fill quality and construction quality of dam. Therefore, the initial impoundment period is a representative period in the core rock-fill dam life cycle. We used the angle forward intersection method to conduct automatic observation of the collimation line. The typical measuring point DB-L5-TP-07 of the collimation line L5 was used as an example of the data analysis.

### 5.1. Analysis of Measured Data

The displacement was obtained by the total station once a day, the water level data was obtained by the water level indicator upstream and downstream of the dam twice a day. The atmospheric pressure was obtained by the barometer once a day. The temperature was obtained by a thermometer once a day, and the rainfall was obtained by a rain gauge once a day. A total of 27 sets of measured data were obtained during the initial storage. To study the relationship between the dependent and independent variables, the following model was established:

(15) $$\delta_{W}=\delta_{H}\;+\;\delta_{T}\;+\;\delta_{T\prime }+\delta_{J}\;+\;\delta_{P}\;+\;\delta_{H\prime }$$

where $\delta_{W}$ is the displacement, which is the dependent variable. $\delta_{H}$ is the upstream water level; $\delta_{T}$ is the temperature; $\delta_{T\prime }$ is the time; $\delta_{J}$ is the rainfall capacity; $\delta_{P}$ is the atmospheric pressure, and is the downstream water level; these are independent variables.

Equation (15) is rewritten as follows:

(16) $$\begin{array}{l} D_{\left(n\right)}=\alpha_{0}\cdot (H_{\left(n\right)}-H_{0})+\alpha_{1}\cdot (T_{\left(n\right)}-\bar{T})+\alpha_{2}\cdot In(T_{\left(n\right)}^{\prime }-T_{0}^{\prime })\\ +\alpha_{3}\cdot J_{\left(n\right)}+\alpha_{4}\cdot (P_{\left(n\right)}-\bar{P})+\alpha_{5}\cdot \left(H_{\left(n\right)}^{\prime }-H_{0}^{\prime }\right)+\alpha_{6} \end{array}$$

Considering Equation (16), $D_{\left(n\right)}$ is the displacement at time n (mm). $H_{\left(n\right)}$ and $H_{0}$ are the upstream and reference upstream water levels (m), respectively. $T_{\left(n\right)}$ and $\bar{T}$ are the temperature at time n and average temperature (°C), respectively. $T_{\left(n\right)}^{\prime }$ is the current time, and $T_{0}^{\prime }$ is the time when the reference value is taken. $J_{\left(n\right)}$ is the rainfall at time n (mm). $P_{\left(n\right)}$ and $\bar{P}$ are the atmospheric pressure at time n and the average atmospheric pressure (mbar), respectively. $H_{\left(n\right)}^{\prime }$ and $H_{\left(n\right)}^{\prime }$ are the downstream water levels at time n and when the reference value is taken (m), respectively. $\alpha_{0},\alpha_{1},\alpha_{2},\alpha_{3},\alpha_{4},\alpha_{5}$ and $\alpha_{6}$ are undetermined coefficients.

A multiple linear regression was used to calculate the model parameters and variance inflation factor (VIF) [32], and the results are shown in Table 1.

VIF = variance inflation factor. VIF is an index to judge whether there is multicollinearity. The definition is as follows:

$$VIF_{i}=\frac{1}{1-R_{i}^{2}}$$

where, $R_{i}^{2}$ is the complex correlation coefficient.

As shown in Table 1, the coefficient of the time factor was negative, which is inconsistent with the observations. Additionally, the VIF was 101.886 > 10, indicating the existence of multicollinearity between the independent variables and the failure of multiple linear regression modeling. We used the improved partial least squares (IPLS) method, described in Section 4, to obtain the following regression equation:

(17) $$\begin{array}{l} D_{\left(n\right)}=1.701*(H_{\left(n\right)}-H_{0})+0.799*(T_{\left(n\right)}-\bar{T})\\ +584.957*In(T\prime_{\left(n\right)}-T\prime_{0})-0.190*J_{\left(n\right)}\\ +0.531*(P_{\left(n\right)}-\bar{P})-0.716*(H\prime_{\left(n\right)}-H\prime_{0})+\;42.082 \end{array}$$

A positive regression coefficient indicates a positive correlation. Equation (17) shows that the displacement was positively correlated with the upstream water level, time and temperature. The process line and relevant behaviors are elaborated in the next section.

### 5.2. IPLS Regression Coefficient Analysis

**Figure 7:**
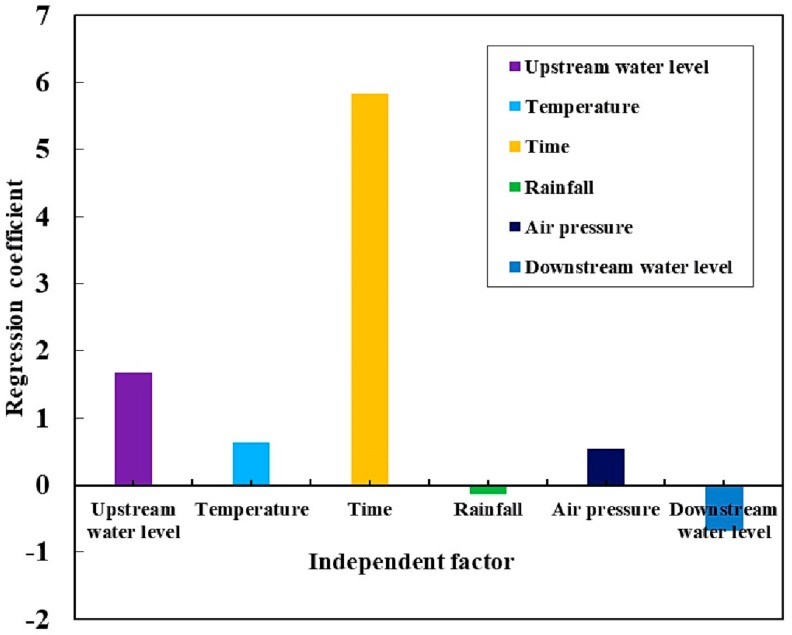
Histogram of regression coefficients. The coefficient value of time divided by 100.

Based on the regression coefficients for each factor obtained by the IPLS, a histogram of the regression coefficients was drawn, as shown in Figure 7.

**Figure 8:**
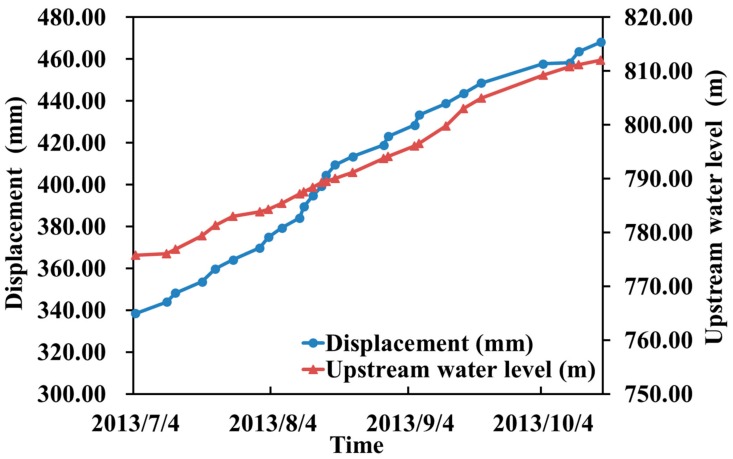
Process line between displacement and upstream water level. During the initial impoundment, the displacement increased with the upstream water level and time.

The histogram shows that there was a strong positive correlation between the independent and dependent variables of the upstream water level and time, but the influencing factors of temperature, rainfall, pressure, downstream water level and dependent variables had a weak correlation, compared with the upstream water level and time influence factors. During the initial storage stage, the displacement of the middle of the downstream rock-fill crest increased with the increase in the upstream water level and time. Figure 8 shows the correlation between displacement and independent variables, further explaining the phenomenon shown in Figure 7.

**Figure 9:**
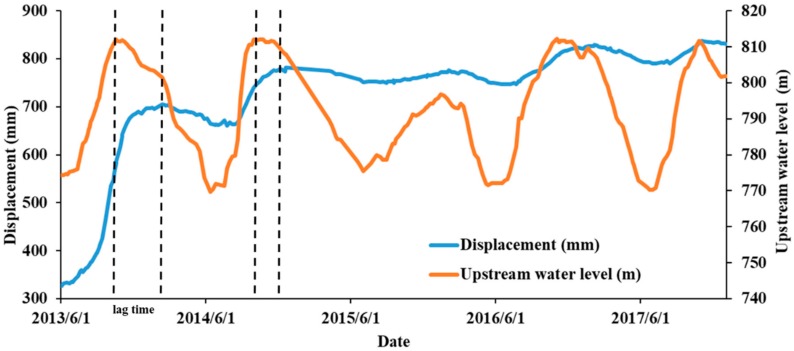
Correlation diagram of displacement and water level changes. The water level change was proportional to the change of the displacement. There is lag time between reservoir level fluctuations and deformation.

We further analyzed the data and compared the relationship between the changes of the upstream water level and the displacement, and the changes are plotted in Figure 9.

### 5.3. Contrast Analysis with a Conventional Method

**Figure 10:**
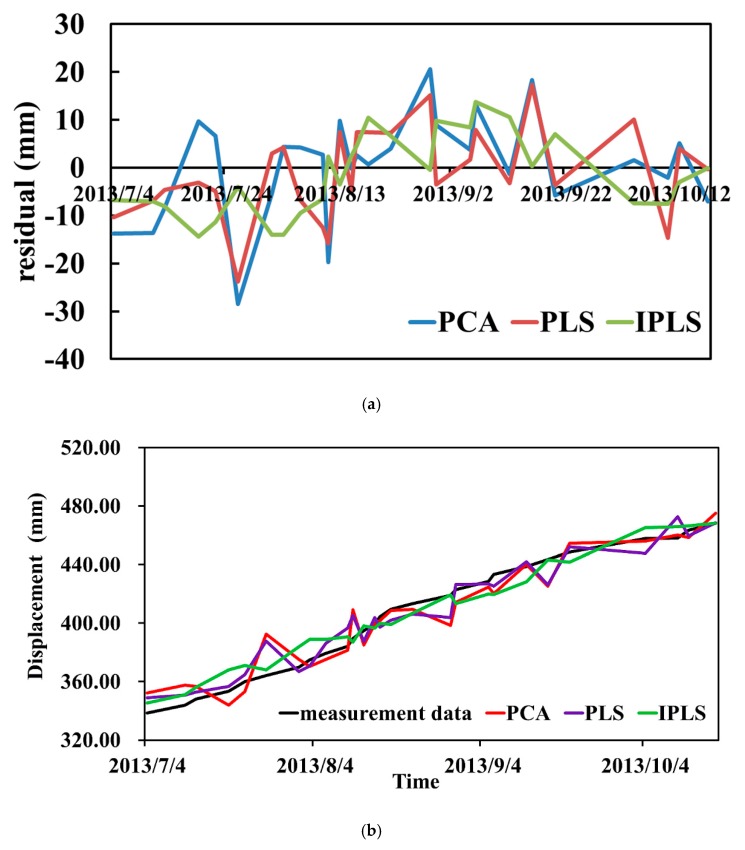
(**a**) Residual comparison diagram; (**b**) Comparison of measured data with the results calculated by the PCA, PLS, and IPLS methods.

A comparison of the accuracy of the observation methods is described in Section 3 and Part 3.2. Here, we compare the data analysis methods. The residuals of the PCA, PLS, and IPLS methods were calculated [33] and are plotted in Figure 10a.

Shown in Figure 10a and the measured data, the PCA residuals fluctuated the most, ranging from −28.48 to 20.62 mm. The PLS residuals fluctuated the second most, ranging from −23.79 to 17.34 mm. The IPLS residuals fluctuated the least, ranging from −14.36 to 13.76 mm. This shows that IPLS was effective and could improve upon the traditional data analysis method.

Meanwhile, combined with the measured data, the displacement calculated using the PCA, PLS, and IPLS methods and the measured data are shown in Figure 10b.

Figure 10b also shows that the measured data were similar to the calculated IPLS results, and the fluctuations were small, which further verified the conclusion drawn in Figure 10a.

### 5.4. Goodness of Fit

**Figure 11:**
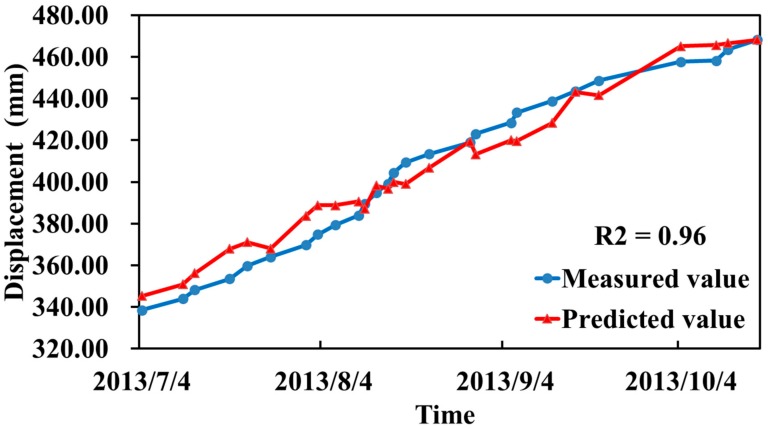
Measured and predicted process lines and the goodness of fit, R^2^.

The goodness of fit was used to measure the quality of the fit [34]. The independent variable factor data were substituted into Equation (17), the predicted value and goodness of fit of the displacement were calculated, and the time series process lines of the measured and predicted values are shown in Figure 11. Meanwhile, the goodness of fit is evaluated with the p values. We use the Statistical Product and Service Solutions (SPSS) tools to calculate p values for PCA, PLS and IPLS. We found that p value for IPLS is 0.543, less than the p value of PCA (0.715) and PLS (0.625). The estimate of the goodness of fit (GoF) is significant.

The goodness of fit, R^2^ = 0.96, indicated that the fitting quality was good and that the model could be used to make predictions. Similarly, the goodness of fit values for the PCA and PLS methods were R_pca_ = 0.92 and R_pls_ = 0.94, respectively. The fitting qualities were also good and can be used to make predictions. However, the IPLS method yielded a better fit.

## 6. Discussion and Conclusions

Improved observation method and improved partial least squares (IPLS) data analysis methods were proposed to overcome the shortcomings of the traditional methods of external deformation monitoring and data analysis of high rock-fill dams. Through a detailed equation derivation and accuracy verification of the proposed angle forward intersection method, we found that the angle forward intersection method exhibits a higher accuracy than the traditional method and can be used for automatic observations. The observation efficiency and accuracy were significantly improved compared with the traditional small-angle method. When the measurement distance was 300 m, the accuracy of measurement was improved by 8.9%. However, when the intersection angle is less than 90°, the accuracy will be low. Thus, we must ensure that the intersection angle is more than 90° by adjusting the installation position of the working basis, safeguarding the best intersection angle is more than 90° and less than 120°.

Previous studies have shown that multicollinearity between independent variables and dependent variables can be addressed by classical methods, such as PCA and PLS. These methods are used for dimensionality reduction in machine learning, and linear independence is achieved by extracting the principal components. However, if the matrix formed by the independent variables contains a large amount of information unrelated to the dependent variables, the implementation of PCA and PLS is difficult. Using the proposed processing method, an orthogonal projection is used to eliminate the independent variable information that is unrelated to the dependent variable, and PLS is applied to the deleted data (Figure 6). Our data showed that IPLS can not only address the issue of multicollinearity in the data analysis, but also improve the interpretation ability of regression coefficients (Figure 7), reduce the fluctuations of the residual error in the prediction, and improve the goodness of fit (Figure 10 and Figure 11).

We used a total station instrument to automatically observe and obtain the data during the initial storage (2013-7-1 through 2013-10-21). Based on the histogram of the IPLS regression coefficients (Figure 7), the upstream water level and time factors were the main factors that affected the displacement. During the initial stage of impoundment, the displacement of the downstream rock-fill increased with the increase in the upstream water level and time. Furthermore, the speed of impoundment also was proportional to the displacement (tabure 9). When the upstream water level changed significantly, the rock-fill body position changed significantly. When the upstream water level changed slowly, the rock-fill body position changed slowly. This relationship is up to the later stages of the dams’ life. To quantify this relationship, we need to use the finite element method to complete the numerical simulation. This conclusion is helpful for the reservoir management department to pay attention to the water level and other key environmental information and provide data support for the safety appraisal of water storage. The automatic observation method and IPLS data processing method in this paper can provide technical support for external deformation monitoring of core wall rock-fill dams. We can try to use the method mentioned in this article in arc arch dams, gravity dams, and other types of waterpower engineering systems.

Here, the observation and data analysis methods of the external deformation monitoring for HCRFDs are explained and deduced in detail. These methods can be applied not only to rock-fill dams, arch dams, gravity dams, and other dam types, but also to bridges, subways, and other projects, and it has great value in practical engineering. However, the method in this paper also has some limitations. During the establishment of the model, the log function was adopted for the time component due to the short initial storage time. When it is long and periodic, it is better to use a sine or cosine function. Additionally, the model can be used to make predictions (Section 5, Part 5.4), which requires knowledge of the value of each influence factor and, thus, independent learning from sample data cannot be carried out to make predictions. Further research on the prediction methods, such as deep learning, can be carried out to improve the accuracy and efficiency of the prediction.

## Figures and Tables

**Table 1:** Parameter estimation of the multiple linear regression model.

Model Parameter	Coefficient	VIF
Constant	1612.90	
Upstream water level	13.33	99.47
Temperature	−3.06	1.16
Time	−2325.03	101.88
Rainfall	−0.67	1.20
Air pressure	−2.22	1.74
Downstream water level	3.66	1.22
